# Efficient one-pot synthesis, molecular docking and in silico ADME prediction of bis-(4-hydroxycoumarin-3-yl) methane derivatives as antileishmanial agents

**DOI:** 10.17179/excli2015-244

**Published:** 2015-08-10

**Authors:** Zahid Zaheer, Firoz A. Kalam Khan, Jaiprakash N. Sangshetti, Rajendra H. Patil

**Affiliations:** 1 Department of Pharmaceutical Chemistry, Y. B. Chavan College of Pharmacy, Dr. Rafiq Zakaria Campus, Aurangabad 431 001(MS), India; 2 Department of Biotechnology, Savitribai Phule Pune University, Pune 411007, (MS), India

**Keywords:** antileishmanial activity, Leishmania donovani promastigotes, molecular docking study, ADME properties, drug likeness, drug score

## Abstract

Bis-(4-hydroxycoumarin-3-yl) methane derivatives **3(a-l)** were synthesized from 4-hydroxycoumarin and substituted aromatic aldehydes using succinimide-*N*-sulfonic acid as catalyst and evaluated for their *in vitro* antileishmanial activity against promastigotes form of *Leishmania donovani*. Compounds **3a** (IC_50_= 155 μg/mL), **3g** (IC_50_= 157.5 μg/mL) and **3l** (IC_50_= 150 μg/mL) were shown significant antileishmanial activity when compared with standard sodium stibogluconate (IC_50_= 490 μg/mL). Also, synthesized compounds **3(a-l)** did not show cytotoxicity against HeLa cell line upto tested concentrations. Further, molecular docking study against *Adenine phosphoribosyltransferase* of *Leishmania donovani *showed good binding interactions. ADME properties were analyzed and showed good oral drug candidate like properties. The synthesized compounds were also shown good drug likeness and drug score values when compared with drugs currently used in therapy. The present study has helped us in identifying a new lead that could be exploited as a potential antileishmanial agent.

## Introduction

Leishmaniasis is parasitic disease caused by different species of the genus Leishmania protozoan and transmitted to humans by the bite of female phlebotomine sand fly (Chappuis et al., 2007[5]). According to WHO estimates, there are at least 3-5 million clinical cases among the 12 million infected individuals are living in endemic areas (Desjeux, 2001[9]). Visceral leishmaniasis (VL or Kala-azar) is the most devastating form of leishmaniasis and caused by the invasion of the reticuloendothelial system (spleen, liver and bone marrow) by the haemo flagellate protozoan parasite *Leishmania donovani *(*L. donovani*) (Ashford et al., 1992[3]). VL causes hepatosplenomegaly, fever, and weight loss and could be fatal if left untreated (Richard and Werbovetz, 2010[30]). The disease is widely distributed in the Indian subcontinent and South-West Asia (Sundar, 2001[40]). Also, there are growing numbers of reports of Leishmania/human immunodeficiency virus (HIV) co-infections across the world. Leishmania-HIV co-infection has been an increasing problem in countries such as Ethiopia, Sudan, Brazil and India where both infections are becoming more and more prevalent (Cruz et al., 2006[7]; Desjeux et al., 2001[10]). 

The primary chemotherapy of leishmaniasis has been based on the use of pentavalent antimonial drug like sodium stibogluconate (SSG) (Santos et al., 2008[34]). SSG was the most effective antileishmanial drug; however, over the years, its efficacy declined and unfortunately developed resistance in most of the areas where failure rates of up to 65 % have been reported (Croft et al., 2006[6]). Furthermore, the long course of treatment with SSG often causes side effects such as myalgia, pancreatitis, cardiac arrhythmia and hepatitis leading to the reduction or cessation of treatment (Rajiv et al., 2012[29]). Second line drugs include pentamidine and amphotericin B, but these drugs have not experienced widespread use due to the severe toxicities and high costs (Cagnoni, 2002[4]). Up to now, no vaccine has been approved for human use (Grenfell et al., 2010[14]; Noazin et al., 2008[26]). Therefore, there is an urgent need to speed up the development of new, inexpensive, effective and safe drugs for the treatment of leishmaniasis and discovering of new lead compounds for this disease is a pressing concern for global health programs.

**Figure 1:**
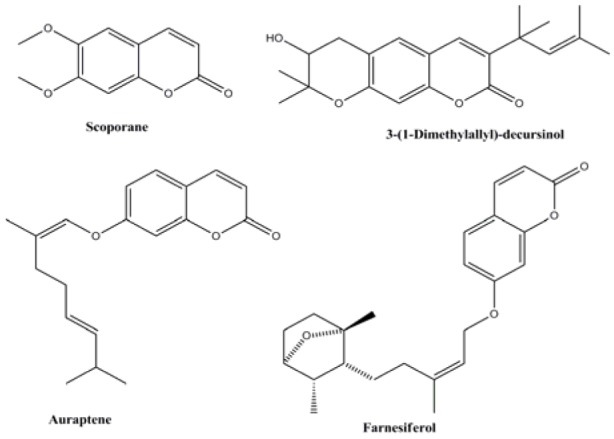
Natural coumarins effective against promastigote form of Leishmania parasite

Coumarins are one of the best known oxygenated heterocycle and present as a structural motif in numerous natural products. Compounds containing coumarin moiety exhibit wide range of biological activities including antiviral (Lee et al., 1998[21]), anticoagulant (Jung et al., 2001[17]), anti-HIV (Hesse and Kirsch, 2002[15]), antioxidant (Melagraki et al., 2009[23]), and anticancer (Jung et al., 2004[18]) activities. There are several examples of coumarin derivatives like scoporane (Arango et al., 2010[2]), 3-(1-dimethylallyl)-decursinol (Iranshahi et al., 2007[16]), auraptene and farnesiferol (Ferreira et al., 2010[13]) (Figure 1(Fig. 1)) isolated from various families of plants, which have been tested and found to be effective against the promastigote form of Leishmania parasite. Some synthetic coumarins such as 4-arylcoumarins have been reported to exhibit potent activity against *L. donovani *(Pierson et al., 2010[28]).

We have selected our title compounds bis-(4-hydroxycoumarin-3-yl) methanes for development and assessment of antileishmanial activity against *L. donovani*. The compounds have been reported to exhibit activities like antioxidant activity (Singh et al., 2010[39]) and antimicrobial activity (Tangmouo et al., 2005[42]) but have not previously been tested against any Leishmania species. In recent years, succinimide-*N*-sulfonic acid as a catalyst has drawn much interest in different organic reactions due to its experimental simplicity (Shirini and Khaligh, 2012[36][38], 2013[35]). To our best knowledge, there is no report in the literature on the preparation of bis-(4-hydroxycoumarin-3-yl) methanes using succinimide-*N*-sulfonic acid as catalyst. 

Due to the lack of effective antileishmanial drugs and in continuation of our earlier work to identify active compounds (Khan et al., 2015[20]; Sangshetti et al., 2014[31][33], 2015[32]), we decided to synthesize and test the efficiency of bis-(4-hydroxycoumarin-3-yl) methanes **3(a-l)** for antileishmanial activity against the promastigote form of *L. donovani* parasite. We had also tested the cytotoxicity study of synthesized compounds against HeLa cell lines. The computational parameters like docking study, ADME prediction, drug likeness and drug score of synthesized compounds were also performed. The results suggest that the compounds could be exploited as an antileishmanial drug.

## Materials and Methods

### Experimental

**Figure 2:**
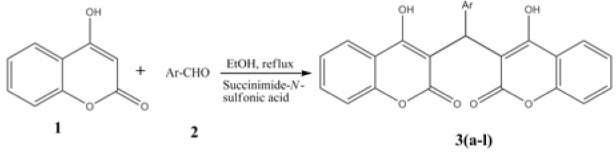
Synthesis of bis-(4-hydroxycoumarin-3-yl) methanes 3(a-l)

4-Hydroxycoumarin, substituted aromatic aldehydes and solvents were obtained from Sigma/Avra synthesis and used without further purification. The synthetic protocols employed for the synthesis of bis-(4-hydroxycoumarin-3-yl) methanes **3(a-l)** are presented in Figure 2(Fig. 2). The homogeneity of the compounds was monitored by ascending thin layer chromatography (TLC) on silica gel-G (Merck) coated aluminum plates, visualized by iodine vapor. The melting points were determined in open capillary tubes. ^1^H NMR and ^13^C NMR spectra were recorded on 300MHz BRUKER spectrometer and 100MHz BRUKER spectrometer, respectively. Chemical shifts are reported in parts per million (ppm), using TMS as an internal standard. Mass spectra were taken with Micromass-QUATTRO-II of WATER mass spectrometer. Elemental analyses (C, H, and N) were undertaken with a Shimadzu's FLASHEA112 analyzer and all analyses were consistent with theoretical values (within 0.4 %).

#### General procedure for the synthesis of bis-(4-hydroxycoumarin-3-yl) methanes 3(a-l)

A mixture of a 4-hydroxycoumarin (2 mmol), aldehydes (1 mmol) was refluxed in ethanol (15 ml) using succinimide-*N*-sulfonic acid (10 mol %) as catalyst for 10-15 min. After completion of the reaction as monitored by TLC analysis, the solid formed was filtered and dried. The crude product was crystallized by using isopropyl alcohol (IPA) to afford the pure product. All the compounds **3(a-l)** were prepared similarly by treating 4-hydroxycoumarin with corresponding aldehydes.

#### 3,3'-(Phenylmethylene)bis(4-hydroxy-2H-chromen-2-one) (3a)

White solid; ^1^H NMR (300 MHz, DMSO-d6) *δ*: 16.39 (1H, s, OH), 7.78−7.39 (8H, m, aromatic H), 7.32-7.10 (5H, m, aromatic H), 6.19 (1H, s, CH), 5.15 (1H, s, OH); ^13^C NMR (100 MHz, DMSO-d6) *δ*: 162.70, 160.90, 152.29, 141.60, 131.81, 130.30, 128.69, 127.88, 125.95, 123.62, 118.22, 116.90, 100.79, 36.71; ES-MS *m/z*: 413.10 [M+H^+^]; Elemental Analysis for C_25_H_16_O_6_. Calcd.: C, 72.81; H, 3.91; Found: C, 72.83; H, 3.90.

#### 3,3'-((4-Chlorophenyl)methylene)bis(4-hydroxy-2H-chromen-2-one) (3b)

White solid; ^1^H NMR (300 MHz, DMSO-d6) *δ*: 16.32 (1H, s, OH), 7.84−7.42 (8H, m, aromatic H), 7.37-7.17 (4H, m, aromatic H), 6.24 (1H, s, CH), 5.20 (1H, s, OH); ^13^C NMR (100 MHz, DMSO-d6) *δ*: 168.80, 161.90, 152.50, 142.50, 131.30, 130.55, 128.70, 127.22, 125.46, 123.52, 118.65, 116.30, 100.19, 35.79; ES-MS *m/z*: 447.06 [M+H^+^]; Elemental Analysis for C_25_H_15_ClO_6_. Calcd.: C, 67.20; H, 3.38; Found: C, 67.24; H, 3.36.

#### 3,3'-((2,6-Dichlorophenyl)methylene)bis(4-hydroxy-2H-chromen-2-one) (3c) 

White solid; ^1^H NMR (300 MHz, DMSO-d6) *δ*: 16.30 (1H, s, OH), 7.81−7.40 (8H, m, aromatic H), 7.35-7.16 (3H, m, aromatic H), 6.29 (1H, s, CH), 5.18 (1H, s, OH); ^13^C NMR (100 MHz, DMSO-d6) *δ*: 164.32, 160.88, 153.19, 138.50, 135.77, 128.50, 127.54, 126.82, 125.44, 123.68, 118.45, 116.28, 103.29, 26.79; ES-MS *m/z*: 481.28 [M+H^+^]; Elemental Analysis for C_25_H_14_Cl_2_O_6_. Calcd.: C, 62.39; H, 2.93; Found: C, 62.41; H, 2.92.

#### 3,3'-((4-Fluorophenyl)methylene)bis(4-hydroxy-2H-chromen-2-one) (3d) 

White solid; ^1^H NMR (300 MHz, DMSO-d6) *δ*: 16.33 (1H, s, OH), 7.41−7.25 (8H, m, aromatic H), 7.17-7.05 (4H, m, aromatic H), 6.09 (1H, s, CH), 5.18 (1H, s, OH); ^13^C NMR (100 MHz, DMSO-d6) *δ*: 167.15, 162.19, 159.90 151.92, 140.00, 131.60, 128.55, 125.40, 123.55, 118.55, 116.36, 115.30, 100.29, 34.95; ES-MS *m/z*: 431.09 [M+H^+^]; Elemental Analysis for C_25_H_15_FO_6_. Calcd.: C, 69.77; H, 3.51; Found: C, 69.80; H, 3.50.

#### 3,3'-((4-Methoxyphenyl)methylene)bis(4-hydroxy-2H-chromen-2-one) (3e)

White solid; ^1^H NMR (300 MHz, DMSO-d6) *δ*: 16.33 (1H, s, OH), 7.66-7.31 (8H, m, aromatic H), 7.25−7.09 (4H, m, aromatic H), 6.31 (1H, s, CH), 5.24 (1H, s, OH), 3.60 (3H, s, OCH_3_); ^13^C NMR (100 MHz, DMSO-d6) *δ*: 164.85, 161.49, 157.19, 152.17, 136.46, 129.84, 128.69, 125.13, 123.48, 118.50, 116.11, 114.32, 103.21, 58.85, 39.79; ES-MS *m/z*: 443.11 [M+H^+^]; Elemental Analysis for C_26_H_18_O_7_. Calcd.: C, 70.58; H, 4.10; Found: C, 70.60; H, 4.09.

#### 3,3'-((3,4-Dimethoxyphenyl)methylene)-bis(4-hydroxy-2H-chromen-2-one) (3f)

White solid; ^1^H NMR (300 MHz, DMSO-d6) *δ*: 16.37 (1H, s, OH), 7.75-7.37 (8H, m, aromatic H), 7.26−7.15 (3H, m, aromatic H), 6.20 (1H, s, CH), 5.13 (1H, s, OH), 3.72 (3H, s, OCH_3_), 3.64 (3H, s, OCH_3_); ^13^C NMR (100 MHz, DMSO-d6) *δ*: 162.80, 158.50, 152.50, 149.70, 146.80, 135.93, 128.80, 125.66, 122.83, 119.98, 116.35, 114.81, 112.43, 101.53, 56.58, 36.72; ES-MS *m/z*: 473.12 [M+H^+^]; Elemental Analysis for C_27_H_20_O_8_. Calcd.: C, 68.64; H, 4.27; Found: C, 68.62; H, 4.28.

#### 3,3'-((2,4-Dimethoxyphenyl)methylene)bis(4-hydroxy-2H-chromen-2-one9 (3g) 

White solid; ^1^H NMR (300 MHz, DMSO-d6) *δ*: 16.36 (1H, s, OH), 7.70-7.35 (8H, m, aromatic H), 7.24−7.18 (3H, m, aromatic H), 6.42 (1H, s, CH), 5.11 (1H, s, OH), 3.91 (3H, s, OCH_3_), 3.74 (3H, s, OCH_3_); ^13^C NMR (100 MHz, DMSO-d6) *δ*: 163.55, 161.10, 159.19, 156.72, 146.53, 131.45, 129.10, 125.28, 123.38, 118.28, 116.36, 112.36, 106.85, 101.03, 59.21, 55.48, 30.89; ES-MS *m/z*: 473.45 [M+H^+^]; Elemental Analysis for C_27_H_20_O_8_. Calcd.: C, 68.64; H, 4.27; Found: C, 68.65; H, 4.26.

#### 3,3'-((2,5-Dimethoxyphenyl)methylene)bis(4-hydroxy-2H-chromen-2-one) (3h) 

White solid; ^1^H NMR (300 MHz, DMSO-d6) *δ*: 16.34 (1H, s, OH), 7.65-7.39 (8H, m, aromatic H), 7.22−7.10 (3H, m, aromatic H), 6.39 (1H, s, CH), 5.38 (1H, s, OH), 3.86 (3H, s, OCH_3_), 3.42 (3H, s, OCH_3_); ^13^C NMR (100 MHz, DMSO-d6) *δ*: 166.19, 161.87, 156.46, 152.58, 149.29, 130.28, 125.10, 123.37, 123.38, 122.70, 118.25, 116.13, 114.73, 112.21, 104.59, 100.89, 60.63, 55.27, 33.01; ES-MS *m/z*: 473.19 [M+H^+^]; Elemental Analysis for C_27_H_20_O_8_. Calcd.: C, 68.64; H, 4.27; Found: C, 68.66; H, 4.27.

#### 3,3'-((3,4,5-Trimethoxyphenyl)methylene)bis(4-hydroxy-2H-chromen-2-one) (3i)

Light yellow solid; ^1^H NMR (300 MHz, DMSO-d6) *δ*: 16.34 (1H, s, OH), 7.80-7.41 (8H, m, aromatic H), 7.31−7.12 (2H, m, aromatic H), 6.28 (1H, s, CH), 5.41 (1H, s, OH), 3.68 (3H, s, OCH_3_), 3.51 (3H, s, OCH_3_) 3.12 (3H, s, OCH_3_); ^13^C NMR (100 MHz, DMSO-d6) *δ*: 165.33, 160.98, 156.84, 152.04, 136.50, 132.57, 128.39, 125.43, 122.32, 119.04, 116.21, 106.40, 102.31, 64.20, 56.87, 37.42; ES-MS *m/z*: 503.13 [M+H^+^]; Elemental Analysis for C_28_H_22_O_9_. Calcd.: C, 66.93; H, 4.41; Found: C, 66.90; H, 4.42.

#### 3,3'-((4-Hydroxy-3-methoxyphenyl)methylene)bis(4-hydroxy-2H-chromen-2-one ) (3j)

White solid; ^1^H NMR (300 MHz, DMSO-d6) *δ*: 16.37 (1H, s, OH), 7.87-7.56 (8H, m, aromatic H), 7.35−7.27 (3H, m, aromatic H), 6.25 (1H, s, CH), 5.76 (1H, s, OH), 5.35(1H, s, aromatic OH), 3.69 (3H, s, OCH_3_); ^13^C NMR (100 MHz, DMSOd6) *δ*: 166.68, 163.65, 152.24, 148.44, 146.98, 131.59, 128.89, 125.52, 123.84, 122.35, 118.88, 116.38, 115.83, 111.60, 104.21, 55.57, 35.65; ES-MS *m/z*: 459.10 [M+H^+^]; Elemental Analysis for C_26_H_18_O_8_. Calcd.: C, 68.12; H, 3.96; Found: C, 68.18; H, 3.95.

#### 3,3'-((4-Cyanophenyl)methylene)bis(4-hydroxy-2H-chromen-2-one) (3k) 

White solid; ^1^H NMR (300 MHz, DMSO-d6) *δ*: 16.34 (1H, s, OH), 7.73-7.42 (8H, m, aromatic H), 7.31−7.25 (4H, m, aromatic H), 6.31 (1H, s, CH), 5.38 (1H, s, OH); ^13^C NMR (100 MHz, DMSOd6) *δ*: 165.86, 162.46, 154.42, 148.70, 132.10, 128.78, 124.98, 122.21, 120.54, 118.37, 117.43, 115.76, 109.82, 103.07, 36.51; ES-MS *m/z*: 438.19 [M+H^+^]; Elemental Analysis for C_26_H_15_NO_6_. Calcd.: C, 71.39; H, 3.46; N, 3.20; Found: C, 71.42; H, 3.47; N, 3.21.

#### 3,3'-((4-(Dimethylamino)phenyl)methylene)bis(4-hydroxy-2H-chromen-2-one) (3l) 

Yellow solid; ^1^H NMR (300 MHz, DMSO-d6) *δ*: 16.33 (1H, s, OH), 7.80-7.39 (8H, m, aromatic H), 7.30−7.21 (4H, m, aromatic H), 6.48 (1H, s, CH), 5.87 (1H, s, OH), 3.06 (6H, s, CH_3_); ^13^C NMR (100 MHz, DMSOd6) *δ*: 167.30, 163.37, 153.81, 149.07, 133.90, 129.18, 127.19, 125.64, 123.54, 120.18, 116.13, 112.54, 104.11, 41.34, 36.20; ES-MS *m/z*: 456.14 [M+H^+^]; Elemental Analysis for C_27_H_21_NO_6_. Calcd.: C, 71.20; H, 4.65; N, 3.08; Found: C, 71.18; H, 4.67; N, 3.10.

### Biological evaluations

#### In vitro antileishmanial activity

The assay for *in vitro* antileishmanial activity on culture of *L. donovani* promastigotes (NHOM/IN/80/DD8) was carried out in 96-well tissue culture plates. The promastigotes culture was maintained at 22 ^ο^C in modified RPMI 1640 pH 7.4 (without phenol red) with 10 % FCS medium. Drug dilutions were prepared in DMSO and appropriate concentration of each drug was used in triplicate. Plates were incubated at 22 ^o^C for 72 h and evaluated using modified MTT assay, where the conversion of 3-(4,5-dimethylthiazol-2-yl)-2,5-diphenyltetrazolium bromide (MTT) to formazan by mitochondrial enzymes served as an indicator of cell viability and the amount of formazan produced was directly proportional to the number of metabolically active cells. Accordingly, absorbance at 492 nm represented the number of live cells. The concentration that decreased cell growth by 50 % (IC_50_) was determined by graphic interpolation. Pentamidine and sodium stibogluconate were used as standard drugs (Dutta et al., 2005[11]).

#### In vitro cytotoxicity study

Cytotoxic study of the synthesized compounds against HeLa cell line were evaluated by 3-(4,5-dimethylthiazol-2-yl)-2,5-diphenyltetrazolium bromide (MTT) method. HeLa cells were seeded in a 96-well culture plate; 100 mL of a 105 cell/mL suspension in each well in RPMI 1640 supplemented with 10 % FCS culture medium. After incubation at 37 °C in a 5 % CO_2_ incubator for 24 h, test compounds with serial concentration (upto 400 µg/mL) were added. The cells were incubated for 72 h, followed by addition MTT solution (5 mg/mL) to each well and further cultivated for 4 h. The absorbance of each well at 550 nm was determined by a microplate spectrophotometer. The cells were also seen under the microscope (Zeiss, Germany) at 10X magnification (Denizot and Lang, 1986[8]).

### Computational studies

#### Docking study 

Docking study was performed using VLife MDS 4.3 package (Vlifesciences, 2015[43]). With this purpose, crystal structure of *Adenine phosphoribosyltransferase* of *L. donovani* (PDB ID: 1QB8) (Phillips et al., 1999[27]) was obtained from the Protein Data Bank in order to prepare protein for docking study. Docking procedure was followed using the standard protocol implemented in VLife MDS 4.3 package and synthesized compounds **3(a-l)** were docked (GRIP batch docking) against three dimensional structure of *Adenine phosphoribosyltransferase* protein.

#### ADME properties

A computational study of synthesized compounds **3(a-l)** was performed for prediction of ADME properties. In this study, we have calculated molecular volume (MV), molecular weight (MW), logarithm of partition coefficient (miLog *P*), number of hydrogen bond acceptors (n-ON), number of hydrogen bonds donors (n-OHNH), topological polar surface area (TPSA), number of rotatable bonds (n-ROTB) and Lipinski's rule of five (Lipinski et al., 2001[22]) using Molinspiration online property calculation toolkit (Molinspiration, 2015[24]). Absorption (% ABS) was calculated by: % ABS= 109-(0.345×TPSA) (Zhao et al., 2002[44]).

#### Drug likeness and drug score

We have also determined the drug likeness and drug score of synthesized compounds **3(a-l)** and compared with standard drugs (pentamidine, sodium stibogluconate amphotericin B and miltefosine) using OSIRIS property explorer online toolkit (http://www.organic-chemistry.org/prog/peo).

## Results and Discussion

### Chemistry

Succinimide-*N*-sulfonic acid catalyst was prepared as reported previously by the reaction of succinimide with chlorosulfonic acid (Shirini and Khaligh, 2011[37]). In our study, successful synthesis of bis-(4-hydroxycoumarin-3-yl) methanes **(**Figure 2(Fig. 2)) was achieved using succinimide-*N*-sulfonic acid as catalyst in ethanol. For the synthesis of bis-(4-hydroxycoumarin-3-yl) methane derivatives **3(a-l)**, we first optimized the effect of catalyst load. The reaction of 4-hydroxycoumarin (2.0 mmol) **(1) **and benzaldehyde (1.0 mmol) (**2**) in ethanol (15 mL) was used as model reaction (compound **3a**). We used succinimide-*N*-sulfonic acid catalyst at various loads such as 5 mol %, 10 mol %, and 15 mol %. From the result (Table 1(Tab. 1)), it is observed that use of 10 mol % succinimide-*N*-sulfonic acid is more useful giving the product up to 98 % yield. The synthetic protocol was then extended for synthesis of all bis-(4-hydroxycoumarin-3-yl) methane derivatives **3(a**-**l) **using 4-hydroxycoumarin and various substituted aromatic aldehydes. The physical data of the synthesized compounds are presented in Table 2(Tab. 2). (References in Table 2: Entry 3a, 3b, 3d, 3e, 3f, 3k, 3l: Karimian et al., 2013[19]; Entry 3c: Al-Kadasi and Nazeruddin, 2012[1]; Entry 3i: Tabatabaeian et al., 2012[41]) All the reactions proceeded well (10-15 min) in ethanol and gave good yields (90-98 %). The purity of the synthesized compounds was checked by TLC and melting points were determined in open capillary tubes. All synthesized derivatives **3(a-l) **were well characterized by means of ^1^H NMR, ^13^C NMR, Mass, and elemental analysis and spectral data confirmed the proposed structures.

### Biological evaluations

#### In vitro antileishmanial activity

**Figure 3:**
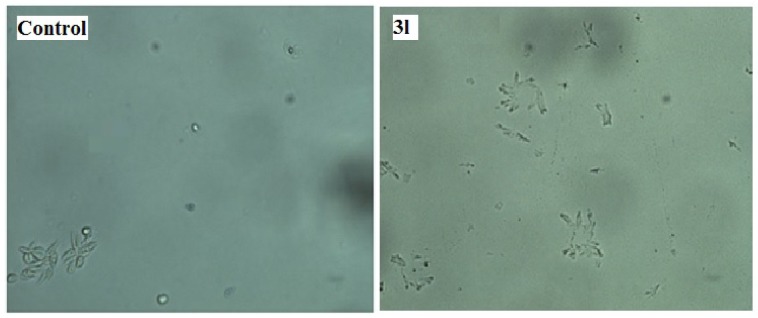
*In vitro* antileishmanial activity of compound 3l against *L. donovani *promastigotes

The assay for *in vitro* antileishmanial activity was carried out on cultures of *L. donovani* promastigotes. The concentration that decreased cell growth by 50 % (IC_50_) was determined by graphic interpolation and data obtained depicted in Table 3(Tab. 3). Compounds **3(a-l)** showed varying degree of antileishmanial activity with IC_50 _ranging between 150 to 320 μg/mL. Amongst all tested compounds **3a, 3g, 3h, 3j** and **3l** were found to be most promising compounds showing IC_50_ value of 155, 157.5, 197.5, 197.5 and 150 μg/mL, respectively. All the synthesized compounds showed better activity than standard sodium stibogluconate (IC_50_= 490 μg/mL) against *L. donovani *promastigotes. A representation of the effect of compound **3l** on the *L. donovani *promastigotes is given in Figure 3(Fig. 3) and revealed that organisms lost its viability as seen by irregular shape morphology of the same.

Structure activity relationship revealed that 4-hydroxycoumarin ring is required for activity. From activity data (Table 3(Tab. 3)), antileishmanial activity mainly depends upon the presence of substituent on phenyl ring. Compound without any substituent on phenyl ring (**3a**) has shown promising activity (IC_50_= 155 μg/mL). Substitution of electron withdrawing group (*-Cl* or *-F*) on phenyl ring **3b**, **3c** and **3d** leads to decrease in activity (IC_50_= 312.5, 320 and 295 μg/mL, respectively). Compounds with *-OCH_3_* group on phenyl ring **3e**, **3f**, **3g**, **3h** and **3i** have shown moderate activity (IC_50_= 157.5 to 275 μg/mL). Compounds with *o-OCH_3_* group on phenyl ring **3g** and **3h** are having good activity as compared to compounds with *m-OCH_3_* and* p-OCH_3_* group **3e**, **3f** and **3i**. Introduction of p-*N(CH_3_)_2_* group on phenyl ring **3l** leads to most potent compound (IC_50_= 150 μg/mL) amongst the synthesized compounds.

#### In vitro cytotoxicity study

**Figure 4:**
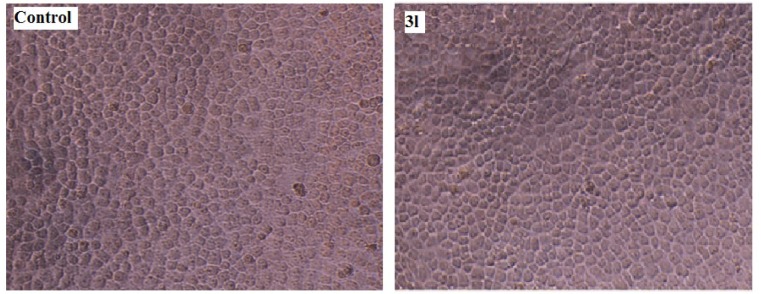
Cytotoxic study of compound 3l against HeLa cell line

Cytotoxic study of the synthesized compounds 3(a-l) against HeLa cell line was evaluated by 3-(4, 5-dimethylthiazol-2-yl)-2, 5-diphenyltetrazolium bromide (MTT) method. None of the synthesized compounds were cytotoxic at concentration upto 400 µg/ mL. A representation of cytotoxic effect of compound 3l on HeLa cell is provided in Figure 4(Fig. 4).

### Computational studies 

#### Docking study

**Figure 5:**
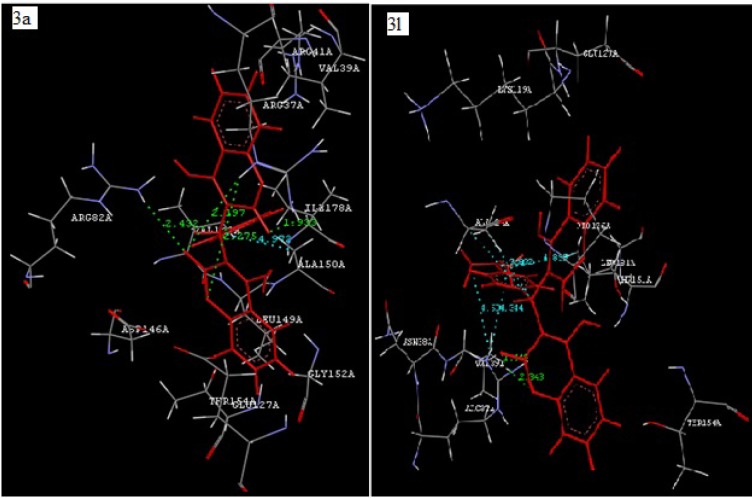
Docking of compound 3a (Left panel) and compound 3l (Right panel) with *Adenine phosphoribosyltransferase* of *L. donovani* (PDB ID: 1QB8). Ligands are shown in red color. Hydrogen bonds are shown in green color. Hydrophobic bonds are shown in sky blue color.

Coumarin derivatives like isopimpinellin are reported to act as antileishmanial by inhibiting the *Adenine phosphoribosyltransferase *enzyme (Napolitano et al., 2003[25]). Molecular docking study of the synthesized compounds **3(a-l)** was performed against *Adenine phosphoribosyltransferase* of *L. donovani* to understand the binding interactions and docking calculation and hydrogen bonds/hydrophobic bonds interactions are shown in Table 3(Tab. 3). All the synthesized compounds had shown good binding energy (-57.51 to -75.19 Kcal/ mol**)** for *Adenine phosphoribosyltransferase*. The results showed that 4-hydroxybiscoumarin core of these compounds held in the active site of enzyme by forming hydrogen, hydrophobic and van der Waals interactions. Amino acids ARG37, ARG82, ALA150 and GLY153 had formed hydrogen bonding and amino acids like ARG37, VAL39, ARG82, ALA124, VAL148, LEU149, ALA150, THR151, GLY152, GLY153, THR154, ALA155 and LEU181 had formed hydrophobic interactions with 4-hydroxybiscoumarin core of compounds. On the basis of activity data and docking result, it is predicted that synthesized compounds **3(a-l)** may have potential to inhibit *Adenine phosphoribosyltransferase*. The interactions of the compound **3a** and **3l **with *Adenine phosphoribosyltransferase* are shown in Figure 5(Fig. 5).

#### ADME properties

A computational study of synthesized compounds **3(a-l)** was performed for prediction of ADME properties. The value obtained is depicted in Table 4(Tab. 4). It is observed that all the synthesized compounds exhibited a good % ABS ranging from 64.03 to 74.19 %. Furthermore, compounds **3b**, **3c** and **3i** violated only one Lipinski's rule of five. None of the active compounds **3a, 3g, 3h, 3j** and **3l** violated Lipinski's parameters. A molecule likely to be developed as an orally active drug candidate should show no more than one violation of the following four criteria: miLog *P* (octanol-water partition coefficient) ≤ 5, molecular weight ≤ 500, number of hydrogen bond acceptors ≤ 10 and number of hydrogen bond donors ≤ 5 (Ertl et al., 2000[12]). All the active synthesized compounds **3a, 3g, 3h, 3j** and **3l** followed the criteria for orally active drug and therefore, these compounds may have a good potential for eventual development as oral agents and may be potentially active new antileishmanial drug candidate.

#### Drug likeness and drug score

In this work, we used the OSIRIS property explorer for calculating the fragment-based drug likeness of all the synthesized compounds **3(a-l)** and other antileishmanial drugs (currently used in therapy) including pentamidine, sodium stibogluconate, amphotericin B and miltefosine (Table 5(Tab. 5)). Our theoretical data showed that all the synthesized compounds (except compound **3k**) presented a drug likeness value higher than the compounds currently used in therapy. In this study, we also verified the drug score, which combines drug likeness, clog *P*, log S, molecular weight and toxicity risks in one value and that may be used to consider the compound overall potential to qualify for a drug. Our data (Table 5(Tab. 5)) showed that all the synthesized compounds (except compound **3k**) presented a very close value to pentamidine, sodium stibogluconate amphotericin B and miltefosine.

## Conclusions

In conclusion, synthesis of bis-(4-hydroxycoumarin-3-yl) methane derivatives** 3(a-l) **has been presented and synthesized compounds were investigated for antileishmanial activity. We have reported succinimide-*N*-sulfonic acid as efficient catalyst for the one-pot synthesis of bis-(4-hydroxycoumarin-3-yl) methanes in ethanol. The products were obtained in excellent yields and the reaction times were significantly short. Compounds **3a**, **3g**, and **3l **were most active for antileishmanial activity when compared with sodium stibogluconate and may serve as a lead compounds for further studies. Molecular docking study showed good binding of these compound to the active site of *Adenine phosphoribosyltransferase* of *L. donovani*. Also, none of the synthesized compounds were cytotoxic to HeLa cell line upto the concentration 400 μg/mL. Furthermore, analysis of the ADME parameters showed good drug like properties and can be developed as oral drug candidate. Drug likeness values of synthesized compounds were better than compounds currently used in therapy. Also, drug score values of compounds were very close to standard drugs (pentamidine, sodium stibogluconate, amphotericin B and miltefosine). Thus, present study helps us in identifying bis-(4-hydroxycoumarin-3-yl) methanes as new lead that could be exploited as a potential antileishmanial agent.

## Acknowledgements

The authors are thankful to the Mrs. Fatima Rafiq Zakaria Chairman Maulana Azad Educational Trust and Principal, Y. B. Chavan College of Pharmacy, Dr. Rafiq Zakaria Campus, Aurangabad 431 001 (M.S.), India for providing the laboratory facility. The authors are also thankful to Bar C. (Department of Zoology, University of Pune) for providing *L. donovani* culture.

## Conflict of interest

The authors declare no conflict of interest.

## Figures and Tables

**Table 1:**
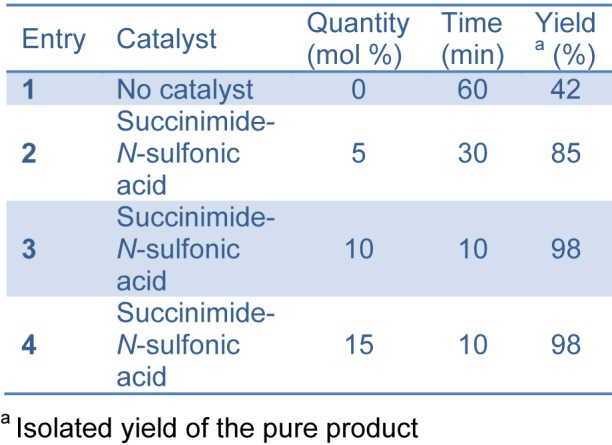
Effect of succinimide-*N*-sulfonic acid (catalyst) loading on yield and reaction time for 3a

**Table 2:**
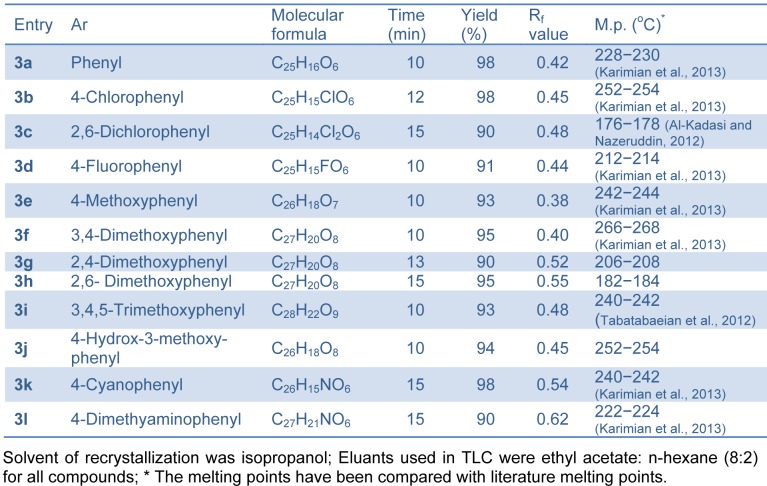
Physical data for bis-(4-hydroxycoumarin-3-yl) methane derivatives 3(a-l)

**Table 3:**
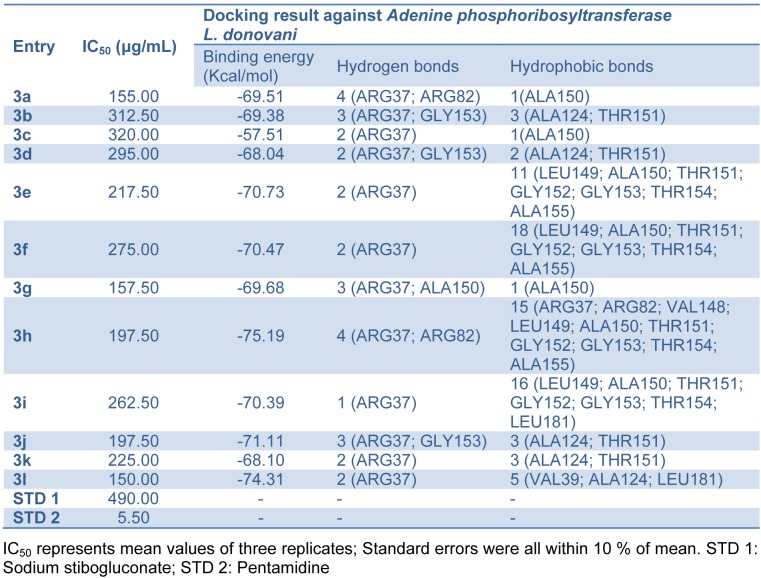
*In vitro* antileishmanial evaluation and molecular docking statistics of bis-(4-hydroxycoumarin-3-yl) methane derivatives 3(a-l)

**Table 4:**
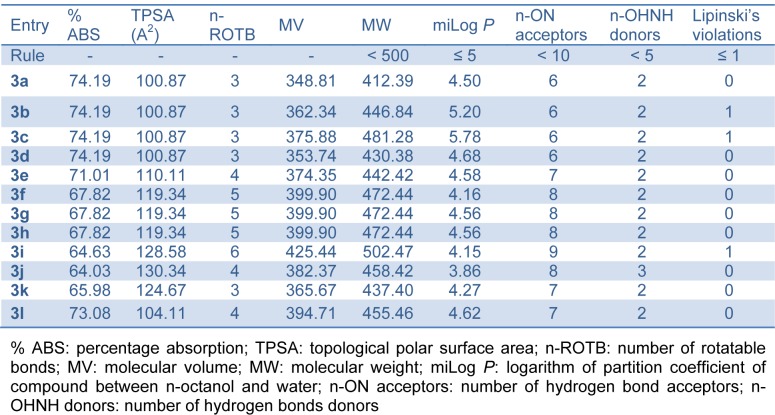
Pharmacokinetic parameters important for good oral bioavailability of bis-(4-hydroxycoumarin-3-yl) methane derivatives 3(a-l)

**Table 5:**
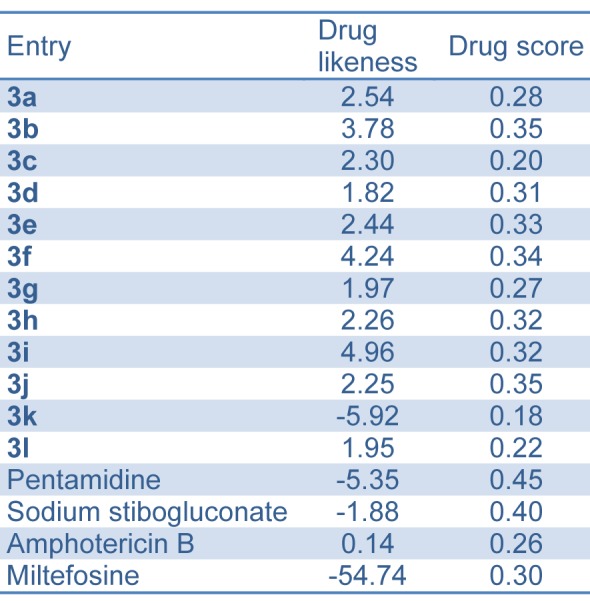
Drug likeness and drug score of bis-(4-hydroxycoumarin-3-yl) methane derivatives 3(a-l) compared to standard drugs
